# Many Cooks in the Kitchen: Iterating a Qualitative Analysis Process Across Multiple Countries, Sites, and Teams

**DOI:** 10.9745/GHSP-D-23-00143

**Published:** 2023-12-22

**Authors:** Lauren Suchman, Serah Gitome, Mandayachepa Nyando, Zachary A. Kwena, Pauline Wekesa, Sarah Okumu, Louisa Ndunyu, Chioma Okoli, Aminat Tijani, Ayobambo Jegede, Ivan Idiodi, Grace Nmadu, Shakede Dimowo, Alfred Maluwa, Lynn Atuyambe, Catherine Birabwa, Phoebe Alitubeera, Betty Kaudha, Agnes Kayego, Tamandani Jumbe, Innocencia Mtalimanja, Janelli Vallin, Elena Sinha, Beth S. Phillips, Dinah Amongin, Elizabeth Bukusi, Kelsey Holt, Martha Kamanga, Jenny Liu, Address Malata, Elizabeth Omoluabi, Peter Waiswa

**Affiliations:** a University of California San Francisco, San Francisco, CA, USA.; b Kenya Medical Research Institute, Nairobi, Kenya.; c Kamuzu University of Health Sciences, Blantyre, Malawi.; d Maseno University, Kisumu, Kenya.; e AkenaPlus Health, Abuja, Nigeria.; f Malawi University of Science and Technology, Limbe, Malawi.; g Makerere University School of Public Health, Kampala, Uganda.; h Karolinska Institute, Stockholm, Sweden.; i Busoga Health Forum, Jinja, Uganda.

## Abstract

Establishing and proving methodological rigor has long been a challenge for qualitative researchers where quantitative methods prevail, but much published literature on qualitative analysis assumes a relatively small number of researchers working in relative proximity. This is particularly true for research conducted with a grounded theory approach. Different versions of grounded theory are commonly used, but this methodology was originally developed for a single researcher collecting and analyzing data in isolation. Although grounded theory has evolved since its development, little has been done to reconcile this approach with the changing nature and composition of international research teams. Advances in technology and an increased emphasis on transnational collaboration have facilitated a shift wherein qualitative datasets have been getting larger and the teams collecting and analyzing them more diverse and diffuse. New processes and systems are therefore required to respond to these conditions. Data for this article are drawn from the experiences of the Innovations for Choice and Autonomy (ICAN) Research Consortium. ICAN aims to understand how self-injectable contraceptives can be implemented in ways that best meet women's needs in Kenya, Uganda, Malawi, and Nigeria. We found that taking a structured approach to analysis was important for maintaining consistency and making the process more manageable across countries. However, it was equally important to allow for flexibility within this structured approach so that teams could adapt more easily to local conditions, making data collection and accompanying analysis more feasible. Meaningfully including all interested researchers in the analysis process and providing support for learning also increased rigor. However, competing priorities in a complex study made it difficult to adhere to planned timelines. We conclude with recommendations for both funders and study teams to design and conduct global health studies that ensure more equitable contributions to analysis while remaining logistically feasible and methodologically sound.

## Abstract (precis)

The authors propose a model for multinational modified grounded theory studies and describe their team's process of collaborating and adapting the model to allow for local needs across countries.

## Teaser

**Key Messages:**

- Multinational teams seeking to conduct modified grounded theory studies should consider adopting a structured, phased approach to data collection and analysis.
- Flexible processes and team structure combined with open communication are key to successfully conducting qualitative analysis across multiple contexts and teams.
- Working toward inclusivity helps all teams respond to rapidly changing study conditions while ensuring more grounded, richer analysis.

**Key Implication:**

- Researchers seeking to conduct complex qualitative analyses with multinational teams should design studies that are structured with built-in flexibility and responsive to local needs while ensuring open lines of communication.

## BACKGROUND

Establishing and proving methodological rigor has long been a challenge for qualitative researchers working in fields where quantitative methods prevail. In an attempt to bring more methodological rigor to their work, Glaser and Strauss^1^ developed grounded theory, which relied on the deep knowledge of a single researcher collecting and analyzing data. Although Glaser and Strauss's original conception of grounded theory has been notably critiqued and reworked over the years,^2^^–^^5^ much of this work still assumes a relatively small number of researchers working in relative proximity—an arrangement that has colonialist roots in anthropologists studying the “other” without including research or individuals from the places under study in the process.^6^ However, advances in technology and an increased emphasis on mixed-methods studies in fields like public health have facilitated a shift wherein qualitative datasets have been getting larger and the teams collecting and analyzing them more diverse and diffuse. Simultaneously, researchers working to decolonize global health are emphasizing more collaborative approaches to data collection and analysis in transnational research that necessitate new processes and systems.^7^ Although there is a body of literature that examines working in teams across the life of a qualitative study,^8^^,^^9^ there is less literature that examines the process, as well as potential opportunities and challenges, associated specifically with analyzing large qualitative datasets across multiple sites, team members, and contexts.

There is not much literature that examines the process, and potential opportunities and challenges, of analyzing large qualitative datasets across multiple sites, team members, and contexts.

Among those who have examined team-based and cross-national qualitative analysis, scholars generally agree that team analysis improves analytical quality by incorporating multiple viewpoints and facilitating reflexivity, which results in a richer and ultimately more accurate final product.^10^^–^^12^ Because qualitative analysis is very time-consuming, some scholars also point out that team-based analysis has the added benefit of saving time and may give more researchers the opportunity to participate in larger studies than they would have otherwise.^13^ However, this body of research on team-based qualitative analysis has largely drawn on the experiences of teams that were either based in the same location or working across locations with similar contexts and access to resources. To our knowledge, the experiences of multinational teams from diverse sociocultural contexts working with large datasets have not been published. To fill this gap, in this article, we examine the analysis process undertaken by a 5-country consortium working to understand how self-injection of the contraceptive subcutaneous depot medroxyprogesterone acetate (DMPA-SC) can be implemented in a way that best meets women's needs within the larger set of contraceptive method options.

## THE INNOVATIONS FOR CHOICE AND AUTONOMY STUDY

This article describes the modified grounded theory qualitative analysis process of the Innovations for Choice and Autonomy (ICAN) study. ICAN aims to understand how self-injection of DMPA-SC can be implemented in a way that best meets women's needs, as defined by women themselves in Kenya, Uganda, Malawi, and Nigeria. From 2020 to 2021, the ICAN consortium conducted a study to deeply understand contraceptive decision-making and to understand for whom self-injection of the contraceptive DMPA-SC may be a powerful method (results forthcoming).

To answer our research questions, ICAN partners in each country conducted a total of 241 (n=∼60/country) semistructured in-depth interviews with women of reproductive age. Women were purposively sampled based on their age (aged 15–19 years and aged 20–45 years), prior contraceptive use or nonuse (to help us deeply understand contraceptive decision-making in general), and previous experience with DMPA-SC (to help us understand for whom self-injection might be a powerful method). In each country, data were collected in 2 study sites (Nairobi and Kisumu metropolitan areas, Kenya; Oyam and Mayuge districts, Uganda; Ntchisi and Mulanje districts, Malawi; and Enugu and Plateau states, Nigeria). Teams used various methods to recruit participants in each site, including working with local community health workers and members of local ICAN Community Advisory Boards to identify potential participants. Some teams (Kenya, Nigeria) elected to hire external data collectors to conduct interviews; core members of the other teams (Malawi, Uganda) conducted interviews themselves. In all cases, data were collected by interviewers who were fluent in appropriate local languages and trained in qualitative research. All participants provided either verbal or written consent to be interviewed, depending on local ethical requirements, and interviews took an average of 1 hour to complete.

ICAN partners are based at the Malawi University of Science and Technology in Malawi; the Makerere University School of Public Health in Uganda; the Kenya Medical Research Institute and Maseno University in Kenya; AkenaPlus Health in Nigeria; and the University of California San Francisco in the United States. The U.S.-based team, as the prime funding recipient, was responsible for overall research design and project administration. Each team based in sub-Saharan Africa was responsible for project administration at the country level while advising on the research approach and collecting all data. ICAN's funder, the Bill & Melinda Gates Foundation, provided both resources and support for cross-country research collaborations and capacity-building.

### Ethical Approval

Ethical approval for the research was obtained from the University of California San Francisco Institutional Review Board (270555, 270747, 270554, 270084); the Kenya Medical Research Institute's Scientific Ethics Review Unit (KEMRI/SERU/CMR/P00136/4013) and Kenyan National Commission for Science, Technology and Innovation (464643); the Makerere University School of Public Health (812) and Uganda National Council of Science and Technology (HS1087ES); the Malawi University of Science and Technology Research Ethics Committee (P.03/2020/007); and the National Health Research Ethics Committee, Nigeria (01/01/2007-25/09/2020).

## THE ICAN QUALITATIVE ANALYSIS PROCESS

### Overall Approach: Modified Grounded Theory

We adopted a grounded theory^1^^,^^2^^,^^4^ approach to this study because our primary aim was to develop theoretical findings related to women's contraceptive decision-making and the relationship between self-injection and contraceptive agency (results forthcoming). We chose this approach over other methods commonly used to develop social theory, such as ethnography^14^ or case studies,^15^ because it best fit the needs of the ICAN study overall. As we have described in detail elsewhere,^16^ the ICAN study has both theoretical and applied goals. Other methods would not have fit within the study timeline while also generating adequate evidence for the study's applied research goals. Still, as grounded theory is a time-intensive process requiring iterative data collection and analysis with potential adjustments to study instruments and sampling structure during fieldwork, we adapted a modified version.^2^ Fully adopting a grounded theory approach proved impractical for our team due to lengthy data collection being inadvisable as a result of security concerns in some sites. In addition, some of our ethical review boards placed constraints on modifications to study instruments, which rendered instruments unmodifiable without lengthy delays.

#### Stage 1: A Structured, Phased Approach to Data Collection and Preliminary Analysis

From February 2021 to January 2022, we conducted data collection with concurrent preliminary analysis in Kenya, Malawi, and Uganda through a systematic approach to iterative data collection and analysis that divided data collection into 4 phases with structured analysis pauses between each phase. The Table summarizes the 4 phases and a fifth phase during which the Nigeria ICAN team conducted data collection without pausing for analysis (described further later). A research director from the U.S.-based team worked with leads from each data collection site to coordinate this initial stage. The number of researchers involved in data collection and preliminary analysis during this phase differed by country, ranging from 5 to 7. Each Africa-based team worked to code and memo about the data from their own country; the U.S. ICAN team provided technical support for and contributed to each country-based process and facilitated cross-country analysis.

**TABLE.** Stage 1 Data Collection and Preliminary Analysis Timeline

	Phase	No. Interviews to Conduct^a^	Total No. Interviews to Be Transcribed (Cumulatively)^a^	Min. No. Transcripts Reviewed^a^	Schedule	Activity^a^
Kenya, Uganda, and Malawi	1	10	10	7	Weeks 1 and 2	4 interviews conducted
					Week 3	Analysis
					Weeks 4 and 5	6 interviews conducted
					Week 6	Analysis
	2	15	25	15	Weeks 7 and 8	15 interviews conducted
					Week 9	Analysis
	3	25	50	30	Weeks 10–12	25 interviews conducted
	4	10	60	40	Week 13	Final rapid analysis
					Weeks 14 and 15	10 interviews conducted
Nigeria	5	60	60	N/A	Weeks 16 and 17	60 interviews conducted

Abbreviation: N/A, not applicable.

a Per country.

In Phase 1 of Stage 1, we started data collection slowly to facilitate initial review of the data. We knew from previous experience that processing data would be relatively slow due to the need to simultaneously transcribe and translate audio recordings of each interview. So, we designed the slow start partially to give transcriptionists adequate time to do their work. Once transcripts were finalized, they were uploaded to a shared cloud storage drive that all team members could access. Phases 2–4 of Stage 1 then moved more quickly to scaffold data collection so that we had time to review data, monitor quality, and conduct preliminary analyses. Keeping with our adapted grounded theory approach, we built in regular pauses throughout. These pauses gave teams time to assess whether data collectors should probe participants further on key themes that required additional evidence or focus on types of women (e.g., adolescents and married women) who were yielding especially interesting or surprising data.

To bypass the extra time needed for transcription at this stage, we drew on the literature on rapid qualitative analysis,^17^ which suggests time-saving strategies for eliminating the use of transcripts^18^ or speeding up transcript production.^19^ In response, we developed a short form that each interviewer could complete after an interview to capture key data points (Supplement 1). These post-interview report forms were programmed into REDCap data collection software. We chose REDCap because it allowed interviewers to complete the forms remotely offline—a critical consideration when conducting data collection in areas with low connectivity—and then immediately transmit their data back to the full team once they were able to connect to the Internet. The simultaneous data collection and analysis process is illustrated in the Figure.

**FIGURE:**
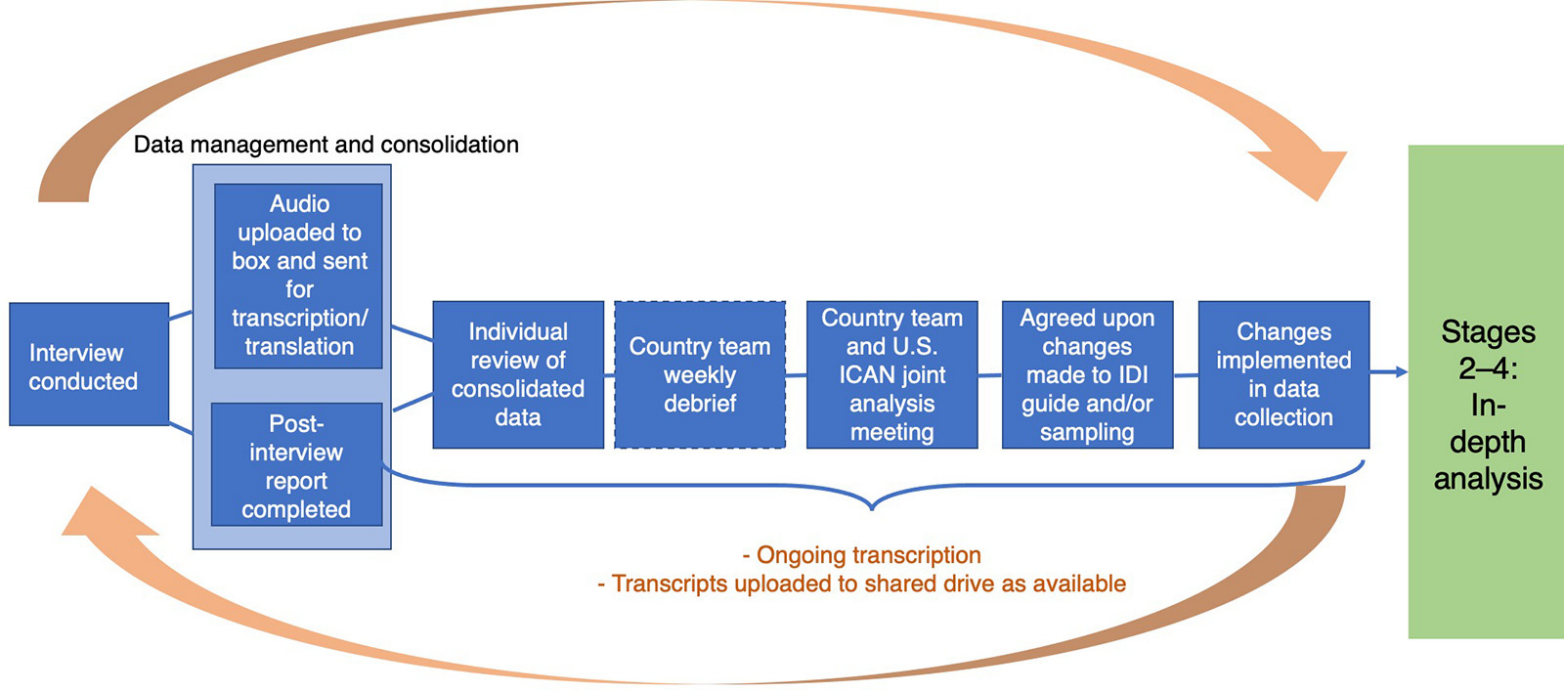
Simultaneous Data Collection and Rapid Analysis Process as Part of Stage 1 in Kenya, Malawi, and Uganda Abbreviations: ICAN, Innovations for Choice and Autonomy; IDI, in-depth interview.

During each analysis pause, team members (including members from U.S. ICAN) were assigned 1–2 full transcripts to review and open code.^20^ Team members also skimmed the post-interview report forms collected since the last pause. In some study countries, ICAN researchers not participating in the qualitative analysis teams also gave input through weekly country team meetings. After this individual and group review, each Africa-based team was joined by U.S. ICAN researchers to discuss preliminary findings and jointly complete a table of initial findings similar to the “data reduction” process described by Watkins.^21^ These tables addressed priority study questions, such as “How do women form contraceptive preferences?” An example table can be found in Supplement 2.

During analysis meetings, key themes began to emerge, such as the critical role that male partners play in women's contraceptive preferences and the importance of accessing health care providers for trustworthy information. Group discussion included a deep exploration of the nuances of, for example, couple dynamics that inform contraceptive decision-making. Identifying these key themes and issues during data collection allowed research assistants to probe more strategically when they returned to data collection after an analysis pause. Analytic teams also used these meetings to discuss and agree on any adjustments to data collection instruments before fieldwork resumed and to begin codesigning a codebook. Notes from the analysis meetings and the post-interview report forms were also used in adjacent ICAN study activities, which are detailed elsewhere.^16^

Identifying key themes and issues during data collection allowed research assistants to probe more strategically when they returned to data collection after an analysis pause.

Data collection and analysis were slightly staggered across countries due to logistical issues and to enable the U.S. ICAN researchers, as facilitators of the cross-country analysis, to participate in each country's preliminary analysis. This staggering also allowed country teams to learn from one another and was particularly beneficial for teams rolling out data collection later to refine field procedures and tools based on prior learnings. Although preliminary analyses were conducted separately for each set of country study data, the U.S.-based researchers facilitated sharing of cross-country findings. To keep meeting times manageable for all, these researchers relayed cross-country findings during individual country team meetings and referred teams to each other to discuss key themes or challenges where appropriate. U.S. ICAN researchers also ensured that edits made to the data collection instruments were updated across countries.

The Nigeria ICAN team chose a modified approach to increase efficiency and began data collection several months after the Kenya, Uganda, and Malawi ICAN teams. While following the same general methodology as other country teams, the ICAN Nigeria team chose to compress data management, consolidation, and preliminary analysis after learning from the experiences of other country teams. They also were able to work with instruments that had gone through several iterations and improvements. With careful planning and piloting, Nigeria ICAN researchers collected data over 2 weeks in September 2021.

#### Stage 2: Standardizing Coding Across Teams

Once data collection was complete in each country, all teams elected to follow a similar process to code the full set of interview transcripts. First, qualitative team researchers participated in an in-depth qualitative analysis training. These trainings were facilitated in each country by team members with expertise in the field with virtual cofacilitation by U.S. ICAN researchers where necessary. This training included an introductory tutorial in the qualitative analysis software package Dedoose. The U.S. ICAN researcher who served as technical lead on the cross-country analysis chose Dedoose for the cross-country analysis because, unlike most qualitative coding programs, it allows for simultaneous coding in real time without the need for manual file exchange and syncing. In this regard, using Dedoose made central management of files relatively straightforward and reduced concerns about version control. However, few others on the research team had experience with Dedoose. During the coding process, some discovered that using a web-based tool was cumbersome for country teams experiencing unstable Internet connectivity and electricity, most notably in Malawi and Nigeria. These factors required researchers to constantly restart the software and slowed progress while adding to frustration.

Each of the coding teams based in Kenya, Uganda, Malawi, and Nigeria standardized coding using a codebook codeveloped across all teams by inductively drawing on findings from the simultaneous data collection and analysis and deductively using the available literature (the final codebook is available in Supplement 3). Although grounded theory typically relies on an inductive approach to analysis, we took this combined approach to meet the applied needs of the study. For example, when developing deductive codes, we mainly relied on the literature related to reproductive empowerment and autonomy^22^^,^^23^ to ensure findings could inform our main analyses of contraceptive decision-making and the relationship between self-injection and contraceptive agency. Although the codebook was standardized as much as possible to allow for cross-national comparison of the data,^24^ it remained flexible to allow for local context to be taken into account.^25^ The codebook largely included index codes to facilitate coding in a large group and some analytic codes designed to address key areas of inquiry as well.

For this stage of the analysis, the Kenya ICAN team developed a process whereby the entire team met as a group and coded a full transcript together over a series of 2-hour virtual sessions. This process was subsequently adopted by the Uganda, Malawi, and Nigeria ICAN teams. U.S. ICAN team members joined these initial virtual sessions to ensure that codes were applied consistently across countries. Although this process was especially time intensive, it allowed researchers to have nuanced conversations about the codebook while standardizing code application across countries. In this way, all team members brought their unique experiences to the analytic process and contributed to a richer, more accurate analysis. After completing the group coding process for 1 full transcript, team members worked in pairs to code 1 additional transcript and compare their coding choices. During this process, teams were able to consult internally, as well as with the ICAN lead of qualitative research, if questions arose.

Coding a full transcript as a team enabled all team members to bring their unique experience to the analytic process and contributed to a richer, more accurate analysis.

Editing the codebook and standardizing code application took 2–4 months for each country team to complete, with some teams greatly delayed in their coding due to COVID-related emergencies. In addition, other components of the larger ICAN study were intensifying in Kenya, Uganda, and Malawi during this period, requiring researchers to divert some time to other study activities. Because adjacent ICAN activities followed a different timeline in Nigeria, the team there finished qualitative data collection and analysis in between other study activities; with all team members dedicated to this activity, coding was completed at the same time as the other country teams despite collecting data much later.

#### Stage 3: Coding Independently and Writing Analytic Memos

Once teams had standardized coding, researchers from each team completed coding a set of individually assigned transcripts using Dedoose. In some cases, such as ICAN Nigeria, the entire team contributed to independent coding. In others, such as ICAN Kenya, additional staff with expertise in qualitative coding were brought in to complete this exercise so that core ICAN researchers could focus their time on other study activities. When additional staff were brought in, teams developed locally appropriate processes to establish standardized code application before coding individually. For example, the ICAN Kenya team worked both internally and with the ICAN qualitative research lead to ensure external coders understood how to use the codebook and to establish standardized code application. Across teams, coders worked on their own timeline with meetings scheduled as needed when questions arose, rather than meeting regularly as in earlier phases of the analysis.

Whether using a version of grounded theory or another approach to qualitative analysis, researchers typically write analytic memos during and after data review. These memos are meant to capture the researcher's interpretation of the data by connecting themes arising from transcript and code review and relating them back to the key research questions.^26^^,^^27^ Although we initially planned to incorporate this type of memo-writing into our coding process using a standardized template in Dedoose, coders found it cumbersome and impractical to switch back and forth between the templates and transcripts and thus rarely used the templates. Instead, some researchers kept their own memos in Microsoft Word documents, which they saved to a shared cloud folder. The benefit of this approach was that it allowed for more flexibility and ease in keeping memos and resulted in accessible notes that other ICAN researchers could easily digest and repurpose for their own analyses without having to download and refer to multiple code reports themselves.

#### Stage 4: Drawing on Code Output and Memos to Answer Multiple Research Questions

Members of the U.S. ICAN team are taking the lead on synthesizing Stage 1 rapid analysis findings and Stage 3 code output into results for our 2 primary cross-country manuscripts on contraceptive decision-making and identifying the relationship between self-injection and contraceptive agency (results forthcoming). For secondary analyses, the Stage 4 analysis process is decentralized to allow multiple members of the consortia to identify and lead individual analyses to address additional research questions for which we collected data. This decentralization is meant to allow us to conduct multiple analyses more efficiently, develop more diverse findings, and disseminate findings more broadly while also giving all team members the opportunity to take leadership roles and experience qualitative analysis from start to finish. Earlier in the ICAN study, all teams contributed to a list of potential manuscripts that they expected might follow from the final analysis. This list was derived from both key study questions and team members' individual research interests. With data fully coded, teams revisited this list, consolidated the list to reduce overlap, and identified priority analyses. To promote participation in writing and leading manuscripts by the full consortium—in particular, early career researchers from each team—ICAN has an authorship secretariat that provides structure and transparency in the process of proposing and leading manuscripts. The secretariat also offers a mentorship program and writing workshops for those seeking writing support. The structure and dynamics of the ICAN authorship secretariat will be explored in a future manuscript.

## DISCUSSION

We have offered a model for conducting modified grounded theory studies across settings using a structured, phased approach. Although we had to adapt the grounded theory approach to account for multiple study needs and the complexities of managing a large, transnational team, we believe we maintained both the spirit and integrity of the methodology. In addition, the collective local knowledge of our diverse researchers allowed us to achieve the deep insights of a lengthier grounded theory study with a limited amount of time to iteratively collect and analyze data. By integrating multiple team members across locations into the initial data collection and analysis process, we were able to draw on a collective brain trust that identified the nuances of key themes early in the research process. This allowed us to probe more strategically throughout the remainder of data collection, generating richer, more accurate findings. It also kept U.S. ICAN researchers apprised of the study's intricacies as they managed analysis across countries.

For other teams looking to adopt such an approach, we note the importance of developing a clear structure within which analysis takes place. Responding to the resource and time limitations that can pose challenges when coding across a team,^28^ some scholars have suggested the use of stage-based coding and standardization, which can create a more rigorous and efficient analytical process.^29^ Indeed, establishing a phased approach to data collection and preliminary analysis, in addition to having a clear schedule and process for the formal analysis, enabled us to thoughtfully develop findings over time while ensuring opportunities for cross-country learnings that strengthened both the analysis process itself and the transferability of findings.

For other teams looking to adopt a similar approach, we note the importance of developing a clear structure within which analysis takes place.

That said, we also emphasize that flexibility, open communication, and local ownership within set structures are important to accommodate the particular needs of different countries and teams. This was most obvious when the Nigeria ICAN team chose not to adopt a phased approach for data collection due to logistical and security concerns. This team was able to collect data over a period of 2 weeks and benefited from feedback on the interview guides and preliminary findings from the other ICAN country teams to help target probing. Maintaining open communication across countries wherein the ICAN Nigeria team was able to voice concerns and propose an alternate plan based on local experience was critical in this process. Many scholars agree that regular, transparent communication among team members increases efficiency as well as intercoder reliability.^9^^,^^30^^,^^31^ This results in a smoother research process and valid findings in addition to strengthening collaboration.^32^

Communication can best be supported by a strong management structure, which clearly defines roles and responsibilities, as well as milestones, keeping the whole team organized and aligned.^33^ However, we concur with Vindrola-Padros and Johnson^17^ that working across teams creates a large administrative burden on all sides, which may be particularly challenging for those working with more limited resources. The U.S. ICAN team was not initially organized for this burden, making centralized process management a challenge. Adding to this problem, as Taylor et al.^34^ found, team members across countries had different percentages of their time allocated to the project, which sometimes made it difficult to coordinate both individuals and teams with multiple competing priorities. With these challenges in mind, the method that Vindrola-Padros et al.^35^ used to share a central study protocol among a number of teams who then conducted research and analysis independently might seem attractive. However, we believe that although a model in which all consortium teams operate with almost complete independence may be more efficient and even necessary in emergencies like the COVID-19 pandemic, this model misses the opportunity to foster and deepen collaboration across teams that we found to be not only rewarding but also integral to creating a truly cohesive multisite study.

Indeed, as other scholars working on team-based analysis have noted, another main benefit of working in teams is the quality that results from a diverse group of people working together to analyze complex data.^10^^,^^11^ We concur but align ourselves with Bird et al.^36^ and suggest bringing an equity lens to this assertion. In our case, we strove to embrace and appreciate diversity across teams. Although overall, we feel we succeeded in enabling a process whereby ICAN members were valued for their individual views and experience, our process of striving for inclusion in the analysis and publication process has not been without challenges. For example, early efforts to put into place authorship guidelines outlining processes for proposing papers and designating co-authors did not go far enough to ensure the inclusion of all relevant team members on large, cross-country papers. Thankfully, open lines of communication facilitated members of the team to highlight these shortcomings and ultimately resulted in a new iteration of more collaboratively developed, robust authorship guidelines. We also faced challenges in ensuring all team members could contribute to their full ability, given uneven coverage of personnel time for all interested members of the analysis teams, highlighting the importance of ensuring all members are adequately supported to participate. In the ICAN consortium, we attempted to do this by developing a mentorship program to give all team members research support they might not otherwise be able to access, as well as revisiting fund allocation and redistributing where possible when inconsistencies between workload and remuneration are identified. Both in-kind and financial resources are crucial for all researchers to do their job well, although adequate financial resources are especially important in the quest for equity.^37^^,^^38^

Finally, as is common in global health research,^39^ the most pressing issue we faced during this process was time. Logistically, coordinating group meetings across multiple time zones and contexts with all team members having varying levels of Internet connectivity and competing demands on their time especially slowed our process of establishing consistency among coders. In this regard, having the qualitative piece of the ICAN study nested within a larger project with a relatively long timeline was beneficial while also posing additional challenges related to time. For example, researchers were staffed on the larger ICAN project beyond the time originally allocated for the qualitative work. As a result, we were able to elongate our timeline to accommodate unexpected events, which we recognize often is not feasible for studies operating on shorter timelines and smaller budgets. Further, the relatively long ICAN timeline gave us more space to be flexible and adaptable to the different contexts in which the study was operating. This was evident in Nigeria, where we were able to adjust the dates of data collection and analysis to align with both local needs and the overall study timeline. However, elongating our timelines caused qualitative coding to overlap with other pieces of the ICAN project, which meant that most researchers had less time to dedicate to coding as time went on. This further delayed the completion of the coding portion of analysis. In this regard, we recognize that our relatively long timeline may limit the applicability of our approach for other studies working with more urgency. In this case, we further emphasize the importance of flexibility when working in complex transnational teams. We found that being able to reallocate some team members' time and simplifying or eliminating processes that weren't working (i.e., the standardized memo templates) allowed us to continue moving forward and complete coding sooner than we would have otherwise.

## RECOMMENDATIONS

Based on the lessons we learned through our own process, we offer the following recommendations for other transnational teams working on complex qualitative analyses and the funders who support this work.

### Funding

- **Funders providing support to institutions not situated in the location(s) where work will take place should require identification of local research partners in the proposal phase**. This was a requirement from ICAN's funder (the Bill & Melinda Gates Foundation), which helped our research consortium form and begin building relationships early. It also preempted the University of California San Francisco (the primary awardee) from sending researchers from the United States to do work best done by local teams.
- **Funders providing support to primary awardees with demonstrated technical capacity to complete the full suite of project activities should require these awardees to commit resources to capacity-building among other research partners where needed**. This requirement from ICAN's funder supported the U.S. ICAN team to budget for activities such as analysis and writing workshops so that more researchers across teams were able to participate in the research process from beginning to end while also gaining transferable skills.
- **Funders should have respect for how long it can take to conduct high-quality research**. This is especially true for qualitative research, which is particularly time-intensive on the analysis side. In the case of ICAN, it was helpful that our program officer had a research background and an accompanying understanding of the qualitative research process. In some cases, funders may have program officers who expect quick turnaround at low cost because they don't have research experience themselves; this risks compromising research quality and the team's ability to collaborate meaningfully.
- **When subcontracts are involved, supporting scopes of work should include adequate detail to ensure each team has sufficient personnel time to fully contribute**. Researchers in global health often work on multiple studies at once, but even for those working on only 1 or 2, complex studies such as ICAN may present competing priorities and strains on researchers' time at multiple points. Clearly envisioning what it will take to achieve each component of the study and articulating this in the scope of work gives all teams the opportunity to participate equitably.

### Working Toward Equitable Collaborations

- **A structured approach to analysis provides a solid foundation on which to develop high-quality findings.** Through our stage-based process, we found that teams were able to iteratively develop findings and fine-tune data collection instruments over time. This allowed more participation in the analysis with diverse perspectives represented and ensured more reliable data.
- **Those leading and managing multisite project teams should remain flexible and adaptable.** It is important for those managing complex, multi-sited projects to be open to shifting plans in line with emerging conditions on the ground (i.e., security issues) or processes that simply don't work for all involved. Further, when power relations are inherently unequal, as is often the case in public health research, it is critical for leaders and managers in positions of power to understand their role in the overall power structure and actively work to change it.^40^^–^^42^ This reflexive and flexible approach to project management can enhance equity while helping study activities run more smoothly.
- **Collaboratively developed authorship guidelines can help facilitate transparency, equity, and inclusion in publication opportunities on large teams.** Authorship is a particularly contested area in global health work with great potential to reinforce unequal power relations.^43^ Particularly on large, multisite teams, proactive and transparent discussions about authorship opportunities can help ensure all are aware of the Contributor Roles Taxonomy (CRediT) authorship model (https://credit.niso.org/) and have equal opportunity to participate in publication opportunities.

## CONCLUSION

As global health evolves and research collaborations become increasingly complex, considerations such as those outlined here will become more pressing and relevant. Our process points to both opportunities and challenges in this space. Although there is no single recipe for success, we believe that “having many cooks in the kitchen” ultimately results in more delicious soup.

## Supplementary Material

GHSP-D-23-00143_supplements_1-2.pdf

GHSP-D-23-00143_supplement_3.xlsx

## Notes

**Peer Reviewed**

**First published online:** November 16, 2023.
