# Supplementary material for: Many Cooks in the Kitchen: Iterating a Qualitative Analysis Process Across Multiple Countries, Sites, and Teams
Source: Glob Health Sci Pract. 2023 Dec 22;11(6):e2300143. doi: 10.9745/GHSP-D-23-00143 (PMC10749643; doi:10.9745/GHSP-D-23-00143)
Supplement: GHSP-D-23-00143_supplements_1-2.pdf [file GHSP-D-23-00143_supplements_1-2.pdf]

Supplement to: Suchman L, Gitome S, Nyando M, et al. Many cooks in the kitchen: iterating a qualitative analysis process across multiple countries, sites, and teams. *Glob Health Sci Pract*. 2023;11(6):e2300143.
https://doi.org/10.9745/GHSP-D-23-00143

SUPPLEMENT 1. ICAN Study Post-interview Report Form

Interview information	
Interview date & time	
Location	
Participant ID #	
Interviewer	

Interview guide	
1. How did the interviewee describe their contraceptive decisions?<br>(Consider when the interviewee started using contraception, how they decided to use contraception, and how they settled on their current contraceptive method.)	
2. How did the interviewee describe the process of procuring contraception?<br>(Consider where the interviewee currently accesses their contraception and if they are satisfied with their current way of accessing contraception.)	
3. What things did the interviewee mention that made it <u>easier</u> to achieve their contraceptive preferences/choices?	
4. What things did the interviewee mention that made it <u>harder</u> to achieve their contraceptive preferences/choices?	
5. What did the interviewee think about self-injection?	
6. Was there anything surprising about the interview?	
7. Did you face any problems or challenges in the interview?	
8. Are there any changes we should consider making to the interview guide? (examples: changing the wording of questions, adding NEW questions or follow-up questions, changing the order of questions)	

Supplement to: Suchman L, Gitome S, Nyando M, et al. Many cooks in the kitchen: iterating a qualitative analysis process across multiple countries, sites, and teams. *Glob Health Sci Pract*. 2023;11(6):e2300143.
https://doi.org/10.9745/GHSP-D-23-00143

SUPPLEMENT 2. ICAN Analysis Meeting Notes – [DATE]

**Research question**	[RESEARCHER 1]	[RESEARCHER 2[	[RESEARCHER 3]
How do women form consciousness about contraceptive rights?			
How do women form contraceptive preferences?			
What influences whether and how women can act on contraceptive rights and preferences?			

Suggested codes:

Suggested changes to the guide:

Advice for interviewers:

Notes for transcribers:

Next steps:
